# β-arrestin1 is an E3 ubiquitin ligase adaptor for substrate linear polyubiquitination

**DOI:** 10.1016/j.jbc.2023.105474

**Published:** 2023-11-21

**Authors:** Chandler J. McElrath, Sara Benzow, Ya Zhuo, Adriano Marchese

**Affiliations:** Department of Biochemistry, Medical College of Wisconsin, Milwaukee, Wisconsin, USA

**Keywords:** arrestin, ubiquitin, E3 ligase, adaptor, AIP4/Itch

## Abstract

G protein–coupled receptor (GPCR) signaling and trafficking are regulated by multiple mechanisms, including posttranslational modifications such as ubiquitination by E3 ubiquitin ligases. E3 ligases have been linked to agonist-stimulated ubiquitination of GPCRs *via* simultaneous binding to βarrestins. In addition, βarrestins have been suggested to assist E3 ligases for ubiquitination of key effector molecules, yet mechanistic insight is lacking. Here, we developed an *in vitro* reconstituted system and show that βarrestin1 (βarr1) serves as an adaptor between the effector protein signal-transducing adaptor molecule 1 (STAM1) and the E3 ligase atrophin-interacting protein 4. *Via* mass spectrometry, we identified seven lysine residues within STAM1 that are ubiquitinated and several types of ubiquitin linkages. We provide evidence that βarr1 facilitates the formation of linear polyubiquitin chains at lysine residue 136 on STAM1. This lysine residue is important for stabilizing the βarr1:STAM1 interaction in cells following GPCR activation. Our study identifies atrophin-interacting protein 4 as only the second E3 ligase known to conjugate linear polyubiquitin chains and a possible role for linear ubiquitin chains in GPCR signaling and trafficking.

βarrestins are multifaceted proteins that play a role in G protein–coupled receptor (GPCR) desensitization, internalization, and signaling (1). βarrestin binding to agonist-activated GPCRs that are phosphorylated by GPCR kinases prevents G protein coupling and links GPCRs for clathrin-mediated endocytosis (1, 2). βarrestins can also serve as signaling scaffolds for multiple effectors thereby promoting signaling (3, 4) and can also assist in GPCR ubiquitination by acting as a bridge between the GPCR and the E3 ubiquitin ligase (5). βarrestins have been reported as E3 ligase adaptors of other GPCRs (5), which was first reported for the β_2_-adrenergic receptor where βarrestin binding to the E3 ubiquitin ligase Nedd4 mediates receptor ubiquitination (6, 7). Their role as adaptor proteins has been determined for other transmembrane proteins, such as receptor tyrosine kinases (8, 9) and ion channels (10, 11, 12). Ubiquitin moieties in these contexts mainly serve as a sorting signal for lysosomal degradation giving rise to long-term attenuation of agonist responsiveness (13), and can also serve as signaling hubs (14).

In addition to transmembrane proteins, there is evidence that βarrestins may also serve as ubiquitination adaptors for cytosolic effector proteins (5, 15). For example, βarr1 binds directly to signal-transducing adaptor molecule 1 (STAM1) (16, 17) and the E3 ubiquitin ligase atrophin-interacting protein 4 (AIP4) (18). These proteins colocalize on early endosomes (16). The disruption of the interaction between βarr1 and STAM1 reduces ubiquitination of STAM1 (19), which is associated with increased lysosomal trafficking and degradation of the chemokine receptor CXCR4 (19, 20). This provides indirect evidence that βarr1 regulates STAM1 ubiquitination.

AIP4 belongs to the homologous to the E6-AP carboxyl terminus domain family of E3 ligases, which typically recognize and bind directly to their ubiquitination targets (21). AIP4 can also bind directly to STAM1 and mediates its ubiquitination upon agonist-stimulation of CXCR4 in cells and is required for the phosphorylation of ERK1/2 (18). Whether βarr1 serves as an ubiquitination adaptor for STAM1 remains to be conclusively determined. Further understanding of STAM1 ubiquitination is important because it has implications for GPCR trafficking and signaling (19, 22).

Here, we reconstituted STAM1 ubiquitination *in vitro* and provide evidence that βarr1 is an adaptor for STAM1 ubiquitination by AIP4. Mass spectroscopy analysis indicates that STAM1 is modified at several lysine residues and with several types of polyubiquitin chain linkages. We also provide evidence that STAM1 is modified with linear polyubiquitin chains at lysine 136 (Lys-136). This is the first time linear polyubiquitin has been associated with an E3 ubiquitin ligase other than linear ubiquitin assembly complex (LUBAC) (23). Our data suggest that βarr1 assists with linear polyubiquitin chain formation on STAM1 at Lys-136 by AIP4. Our study provides new insights into the complexity of ubiquitin chain formation and may expand the roles of linear ubiquitination in cells *via* regulation of GPCR signaling and trafficking.

## Results

**Figure 1:**
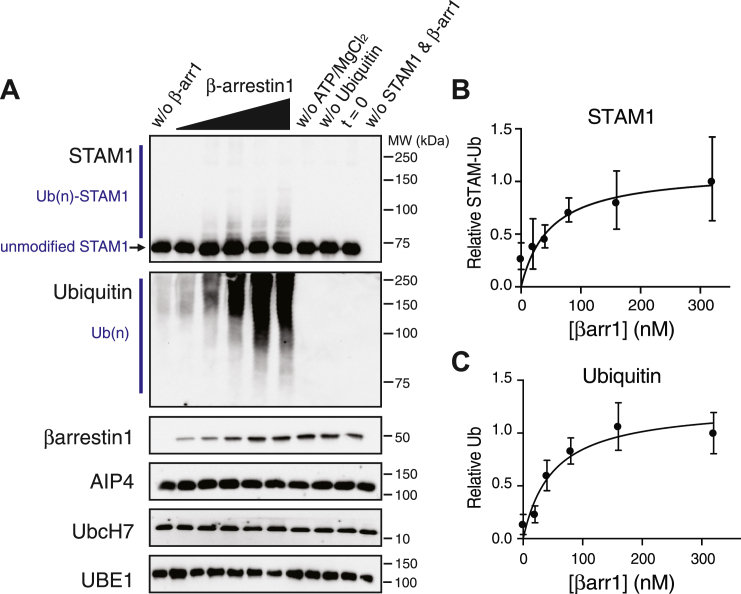
***In vitro* reconstitution of STAM1 ubiquitination by AIP4**. *A*, STAM1 ubiquitination by AIP4 was reconstituted *in vitro* and performed in the presence of increasing concentrations of βarrestin1. Ubiquitination reactions comprised of E1(Ube1, 42 nM), E2 (UbcH7, 350 nM), E3 (AIP4, 48.5 nM), ubiquitin (11.6 μM), DTT (1 mM), ATP (1 mM) plus STAM1 (42 nM), and varying concentrations of βarr1 (0 nM, 20 nM, 40 nM, 80 nM, 160 nM, or 320 nM) in 40 μl. Reactions were incubated for 90 min at 37 °C and terminated with 40 μl 2× sample buffer. Equal volumes were analyzed by 7% SDS-PAGE and immunoblotting with the indicated antibodies. Polyubiquitinated [Ub(n)] and unmodified STAM1 are indicated. Immunoblots are from one representative experiment. STAM1 ubiquitination was quantified from the STAM (*B*) and ubiquitin (*C*) immunoblots using densitometry and shown as line graphs. Data were expressed relative to the signal at 300 nM βarr1 and represent the mean ± S.D. from three independent experiments. Curves were fitted by nonlinear regression, Michaelis–Menten (GraphPad Prism). AIP4, atrophin-interacting protein 4; STAM, signal-transducing adaptor molecule.

Previously, we showed in cell-based assays that the E3 ligase AIP4 interacts with and mediates ubiquitination of STAM1 (18). AIP4 (16) and STAM1 (17) are direct binding partners with βarr1, but whether βarr1 acts as an adaptor for STAM1 ubiquitination by AIP4 remains to be conclusively demonstrated (19). To address this, we reconstituted STAM1 ubiquitination by AIP4 *in vitro*. Ubiquitination of a target protein occurs by an enzymatic cascade involving an ubiquitin-activating enzyme (E1), ubiquitin-conjugating enzyme (E2), the ubiquitin ligase (E3), ubiquitin and ATP/Mg (24). A single E1 enzyme (Ube1) can activate ubiquitin for all ubiquitination reactions (25), and AIP4 is known to pair with the E2 conjugating enzyme UbcH7 (26). STAM1 was incubated with a mixture of E1 (Ube1), UbcH7 (E2), AIP4 (E3), ubiquitin, ATP/MgCl_2_ in the presence or absence of increasing concentrations of βarr1 for 90 min at 37 °C. Reactions were terminated in 2 × sample buffer and analyzed by SDS-PAGE and immunoblotting. Immunoblotting for STAM1 revealed several distinct bands and higher molecular weight bands above unmodified STAM1 consistent with its polyubiquitination (Fig. 1*A*). Importantly, this modification was increased by βarr1 in a concentration-dependent manner (Fig. 1*A*). Although the modification was modest relative to unmodified STAM1, when quantified from three independent experiments, there was a clear dose-dependent increase in STAM1 ubiquitination (Fig. 1*B*). Because STAM1 ubiquitination requires GPCR stimulation (18, 19), and we used inactive βarr1 in these experiments, this likely explains the modest STAM1 ubiquitination (Fig. 1*A*). Based on the size of the ubiquitinated species, this suggests modification by monoubiquitin, multimonoubiquitin and/or short-chain polyubiquitination (Fig. 1*A*), although longer-chain polyubiquitinated species were observed on longer exposures of the anti-STAM1 immunoblot (not shown). The samples were also analyzed by immunoblotting for ubiquitin, which revealed increased longer-chain polyubiquitination in the presence of increasing concentrations of βarr1 (Fig. 1, *A* and *C*). The antiubiquitin antibody likely has higher avidity for longer-chain polyubiquitinated species and is less able to detect mono or multi-monoubiquitin or short-chain polyubiquitinated species. In contrast, the anti-STAM1 antibody might not be able to efficiently detect longer-chain ubiquitinated species due to epitope masking, which might explain why mono or multi-mono and short-chain multiubiquitinated species are easily visible using the anti-STAM1 antibody but not longer-chain polyubiquitinated species. The EC_50_ for βarr1 calculated from the STAM1 (EC_50_ = 48.7 nM, 95% confidence interval = 11.7–166.9) or ubiquitin (49.83 nM, 95% confidence interval = 25.09–95.06) immunoblots were essentially equal. We did not observe ubiquitination of βarr1 or AIP4 in these reactions but cannot rule out the possibility that a portion of the polyubiquitinated species observed with the ubiquitin antibody partially reflects AIP4 and/or βarr1 ubiquitination (Fig. 1*A*). Ubiquitinated species were not detected in reactions without βarr1 and STAM1, suggesting that unconjugated longer-chain polyubiquitinated species are not formed in these reactions (Fig. 1*A*). Polyubiquitination of STAM1 was also detected in the absence of βarr1 and is consistent with cell-based assays that have shown that AIP4 interacts directly with STAM1 and mediates its ubiquitination in cells (18) but represents a small fraction of ubiquitinated STAM1 in our system (Fig. 1*A*). Modified STAM1 or ubiquitinated species were not detected in control reactions that were immediately terminated (t = 0), did not contain ATP/MgCl_2_ or ubiquitin (Fig. 1*A*). Overall, these data indicate that βarr1 enhances STAM1 ubiquitination by AIP4.

**Figure 2:**
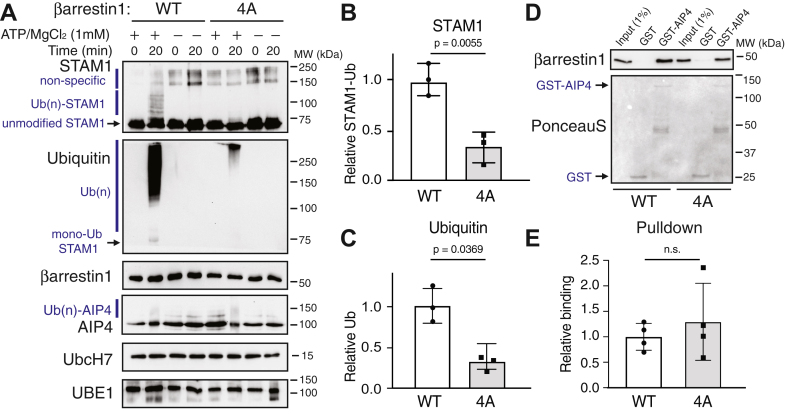
**Analysis of STAM1 ubiquitination with a STAM1 binding-deficient variant of β-arrestin1**. *A*, ubiquitination reactions as described in Experimental procedures were incubated with 40 nM WT β-arr1 (WT) or STAM binding-deficient variant βarr1-4A (4A) in the presence or absence of 1 mM ATP. Reactions were immediately (0 min) terminated or after incubation at 37 °C for 20 min in 2× sample buffer. Reactions were analyzed by 7% SDS-PAGE and immunoblotting with the indicated antibodies. Polyubiquitinated [Ub(n)] and unmodified STAM1 are indicated. Immunoblots are from one representative experiment. STAM1 ubiquitination was quantified from the STAM (*B*) and ubiquitin (*C*) immunoblots using densitometry and shown as *bar graphs*. Data were expressed relative to the signal with WT βarr1 incubated for 20 min and represent the mean ± S.D. from three independent experiments. *D*, binding analysis of AIP4 to WT or 4A βarr1. Equal amounts (500 ng) of purified WT or 4A βarr1 were incubated with equimolar amounts (1 μM) of GST-AIP4 and GST immobilized on glutathione-Sepharose resin. Bound βarr1 was detected by immunoblotting. A small fraction (1%) of purified protein is shown as input. Blots were stained with Ponceau-S to show the level of GST-AIP4 and GST used in the binding reactions. Blots are from one representative experiment. *E*, bound βarr1 was quantified using densitometry, normalized to GST, and shown as a *bar graph*. Data were expressed relative to the WT βarr1 signal and represent the mean ± S.D. from four independent experiments. Data were analyzed by an unpaired *t* test using GraphPad Prism. The adjusted *p* values are indicated. AIP4, atrophin-interacting protein 4; n.s., not significant; STAM, signal-transducing adaptor molecule.

Because βarr1 also interacts directly with AIP4 (16), it is possible that βarr1 directly activates AIP4 (16) rather than serving as an adaptor in these reactions. To explore this possibility, we examined STAM1 ubiquitination in the presence of a βarr1 variant (βarr1-4A) that has reduced affinity for STAM1 (17), but its binding to AIP4 is expected to remain intact since the AIP4 binding site does not overlap with the STAM1 binding site on βarr1 (18). In comparison to reactions containing WT βarr1, those containing βarr1-4A showed reduced ubiquitination of STAM1 as detected by immunoblotting and quantification using densitometry of STAM1 or ubiquitin immunoblots (Fig. 2, *A*–*C*). Nonspecific higher molecular weight immunoreactive bands were detected in all samples, including those incubated without ATP/MgCl_2_, likely because we used a different commercial anti-STAM1 antibody than that used in Figure 1. However, ubiquitinated STAM1 species were clearly visible and importantly were absent in samples incubated without ATP/MgCl_2_ or with βarr1-4A (Fig. 2*A*). These reactions were incubated for 20 min, a time point that showed the greatest reduction in STAM1 ubiquitination by βarr1-4A following a pilot experiment. Interestingly, we detected monoubiquitinated STAM1 species with the ubiquitin antibody, as well as longer-chain polyubiquitinated species in samples incubated with WT βarr1, which were clearly absent or reduced when incubated with βarr1-4A (Fig. 2*A*). A modest amount of AIP4 autoubiquitination was observed in these reactions, which was not impacted by βarr1-4A (Fig. 2) and is consistent with the fact that AIP4 binding to this variant remains intact (Fig. 2, *D*/*E*). Together, these data support a role of βarr1 as an adaptor between STAM1 and AIP4 to facilitate STAM1 ubiquitination.

### Mass spectrometry analysis of STAM1 ubiquitination

**Figure 3:**
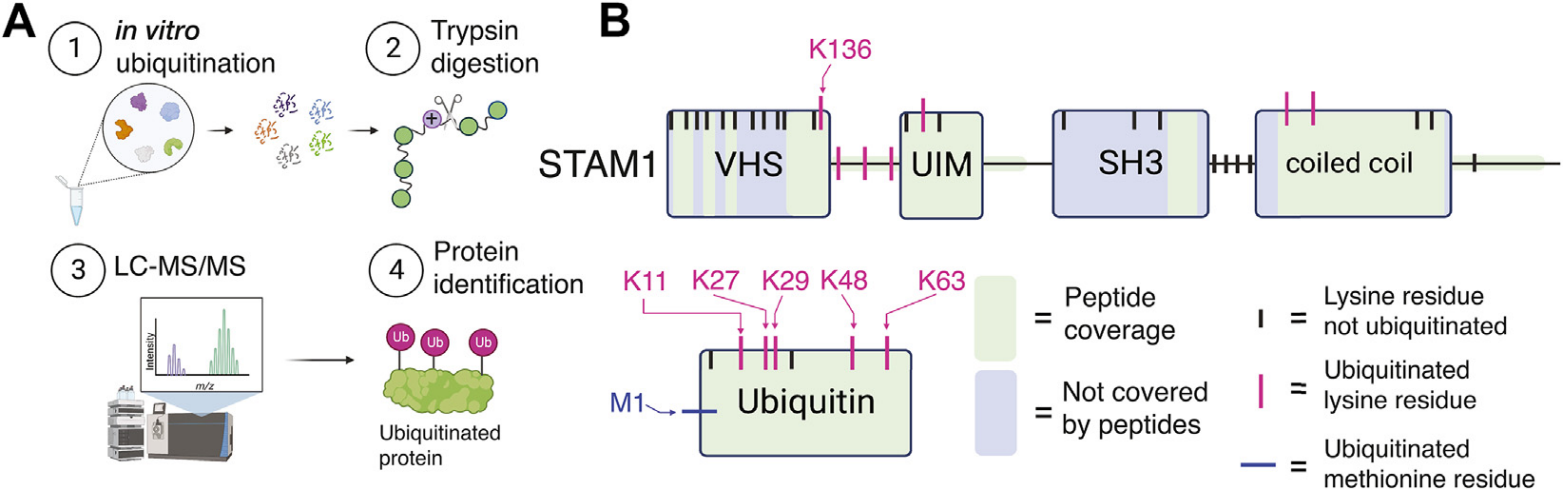
**Mass spectrometry analysis of STAM1 ubiquitination**. *A*, workflow of mass spectrometry analysis of STAM1 ubiquitination. Ubiquitination reactions were as described in Experimental procedures (1), trypsin digested (2), analyzed by liquid chromatography tandem mass spectrometry (LC-MS) (3) and peptide identification (4). *B*, schematic of STAM1 and ubiquitin summary of mass spectrometry results. The *green color* corresponds to peptide coverage of STAM1 or ubiquitin that was observed in the mass spectrometry results, while the *gray color* corresponds to areas that were not covered. STAM has 29 lysine residues (indicated by *ticks*), with seven lysine residues identified to be modified by ubiquitin are indicated by *magenta ticks*. Lysine 136 (K136) is indicated and is located within the VHS (Vps27, HRS, STAM) domain. The UIM (ubiquitin interacting motif), SH3 (Src-homology domain 3) and the coiled-coil domains are shown. Ubiquitin has seven lysine residues (indicated by *ticks*); modified residues are indicated (K11, K27, K29, K48, and K63). The first methionine on ubiquitin was also modified by ubiquitin (M1). The figure was created with BioRender. STAM, signal-transducing adaptor molecule.

To gain a better understanding of the adaptor role of βarr1, we analyzed STAM1 ubiquitination by mass spectroscopy (Fig. 3*A*). Ubiquitination reactions were completed in the presence or absence of βarr1, followed by trypsin digestion, and peptides were analyzed by mass spectrometry for the presence of diGly, which are the last two amino acids of ubiquitin remaining after trypsin digestion at Arg-74 attached to the Lys side-chain (27). In this way, we would expect to identify modified lysine residues on STAM1 or other proteins in our reaction and on ubiquitin itself. We identified a single lysine residue (Lys-136) on STAM1 that was modified by ubiquitin (Fig. 3*B* and Table 1). Additionally, four different types of ubiquitin linkages were identified, including K27, K29, K48, and K63-linked chains (Fig. 3*B* and Table 1), which are linkage types known to be formed by AIP4 (21). We did not detect ubiquitination sites on βarr1 or AIP4 by mass spectrometry. Mass spectrometry data are provided in the File S1.

**Table 1:** List of lysine (K) or methionine (M) residues modified with ubiquitin on STAM1 or ubiquitin

Protein	Ubiquitinated residue	Peptide sequence
STAM1	K136	134-NL**K**EQGVTFPAIGSQAAEQAK-154
	K154	149-AAEQA**K**ASPALVAK-162
	K162	155-ASPALVA**K**DPGTVAN-169
	K170	163-DPGTVAN**K**KEEEDLAK-178
	K185	179-AIELSL**K**EQR-188
	K346	340-GPLIDE**K**LEDIDR-352
	K353	347-LEDIDR**K**HSELSELNV-362
Ubiquitin	K11	7-TLTG**K**TITLEVEPSDTIENVK-27
	K27	16-TITLEVEPSDTIENV**K**AK-29
	K29	26-IQD**K**EGIPPDQQR-38
	K48	43-LIFAG**K**QLEDGR-53
	K63	60-NIQ**K**ESTLHLVLR-72
	M1	76-G**M**QIFVK-6

Shown are the identified peptide sequences with the modified residues bolded and underlined. The amino acid residue numbers relative to the full-length proteins are indicated.

Remarkably, when we analyzed the mass spectrometry data for peptides derived from ubiquitin, we also identified a peptide containing the first seven residues of ubiquitin preceded by two glycine residues, indicative of a linear ubiquitin linkage (Fig. 3*B* and Table 1). This was unexpected since this type of linkage, to our knowledge, has never before been associated with AIP4 and has been previously only associated with one type of E3 ubiquitin ligase (23). In this type of linkage, ubiquitin is fused head-to-tail through a peptide bond between the methionine on one ubiquitin and the terminal glycine residue of another ubiquitin (23). However, it was unclear if STAM1 or any other protein in the reaction was modified by linear ubiquitin chains or whether simply unconjugated linear chains were formed.

To explore this further, we repeated the ubiquitination reactions in the presence or absence of βarr1. However, in contrast to the previous experiment, after the reactions were complete, STAM1-His was purified using Talon resin before it was digested with trypsin and analyzed by mass spectrometry. In this way, we could selectively identify lysine residues on STAM1 that are modified by ubiquitin and possibly also determine the linkage type. Seven lysine residues were identified as ubiquitination sites on STAM1 (Fig. 3*B* and Table 1). We did not detect a difference in ubiquitination sites between reactions that were incubated with or without βarr1 in this experiment. This is not unexpected since AIP4 is also known to bind directly to STAM1 and mediate its ubiquitination (18) and is consistent with STAM1 ubiquitination of STAM1 ubiquitination in the absence of βarr1 (Fig. 1*A*). Notably, one of the residues we identified was Lys-136, which was identified in the previous mass spectrometry experiment. Several AIP4 and βarr1 peptides were also identified (File S1), likely because AIP4 and βarr1 bind directly to STAM1 and were copurified along with His-tagged STAM1.

We also detected modification of Lys residues on ubiquitin, including K27, K29, K48, and K63, which were also identified in the previous mass spectrometry experiment. In addition, we detected a modification at Lys-11, which is a linkage-type that has not been previously associated with AIP4 (21). We also analyzed the mass spectrometry data for peptides derived from ubiquitin. Unlike the previous experiment, we did not observe peptides indicative of a linear ubiquitin linkage. These data suggest that STAM1 is modified by several types of ubiquitin chain linkages, and Lys-136 is a key ubiquitination site on STAM1.

To further investigate whether STAM1 is modified with linear ubiquitin, we performed the ubiquitination reactions in the presence of βarr1 with lysine-free ubiquitin or 0K ubiquitin (0K-Ub), in which all reactive lysine residues have been changed to arginine residues. The C terminus of 0K-Ub is fully functional and can be conjugated to lysine residues on STAM1, and polyubiquitin chains are expected to only form *via* the amine group of the first methionine (Met-1) of ubiquitin because lysine residues are absent. In addition, we used a 0K-Ub in which the N-terminal amine group of Met-1 is modified with a single biotin; therefore, polyubiquitination of any kind cannot form because all reactive amines are absent, although it can be conjugated to lysine residues on STAM1 through its C terminus. After the reactions were complete, STAM1-His was purified using Talon resin before it was digested with trypsin and analyzed by mass spectrometry. With approximately 50% coverage on STAM1, only one ubiquitinated residue, Lys-136, was identified in reactions with 0K-Ub. No ubiquitinated STAM1 peptides were detected in the presence of 0K biotinylated ubiquitin, which does not necessarily suggest that lysine residues were not modified, but rather, suggests that the reaction with this variant ubiquitin was not efficient in our system. Nevertheless, these data confirm that Lys-136 represents a key ubiquitination site on STAM1.

**Figure 4:**
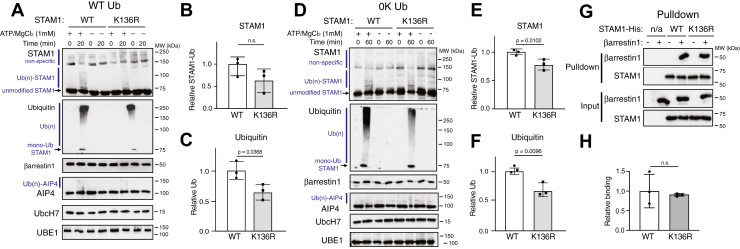
**Evaluation of lysine 136 on STAM1 ubiquitination with WT or lysine-less ubiquitin**. Ubiquitination reactions as described in Experimental procedures were incubated with WT STAM1 (WT) or variant in which lysine 136 has been substituted with an arginine (K136R) and with WT ubiquitin (WT Ub) (*A*) or lysine-less ubiquitin (0K Ub) (*D*). Reactions were performed in the presence of 40 nM βarr1 and with or without 1 mM ATP/MgCl_2_. Reactions were immediately (0 min) terminated in 2× sample buffer or after incubation at 37 °C for 20 min (*A*) or 60 min (*B*). Reactions were analyzed by 7% SDS-PAGE and immunoblotting with the indicated antibodies. Polyubiquitinated [Ub(n)], unmodified and monoubiquitinated (mono-Ub) STAM1 are indicated. Immunoblots are from one representative experiment. STAM1 ubiquitination was quantified from the STAM (*B* and *E*) and ubiquitin (*C* and *F*) immunoblots using densitometry and shown as *bar graphs*. Data were expressed relative to the signal with WT STAM1 and represent the mean ± S.D. from three independent experiments. *G*, purified βarr1 (0.1 μM) was incubated without (empty resin; n/a) or with immobilized STAM1-His (3 μM) WT or K136R variant for 20 min at 37 °C. Complexes were analyzed by immunoblotting. Immunoblots are from a representative experiment. *H*, bound STAM1 normalized to βarr1 was quantified using densitometry and shown as *bar graphs*. Data represent the mean ± S.D. from three independent experiments and are expressed relative to the fraction of STAM1 bound to βarr1. Data were analyzed by an unpaired *t* test using GraphPad Prism. Adjusted *p* values are indicated. n.s., not significant. STAM, signal-transducing adaptor molecule.

Because Lys-136 on STAM1 was repeatedly identified as a ubiquitinated lysine residue *via* mass spectrometry, we substituted Lys-136 to an arginine residue (Arg) and analyzed this variant (K136R) in ubiquitination reactions in the presence of βarr1 using WT ubiquitin (WT-Ub). When compared to wildtype STAM1, ubiquitination of the K136R variant was reduced albeit modestly (Fig. 4*A*). Quantification of either STAM (Fig. 4*B*) or ubiquitin (Fig. 4*C*) immunoblots using densitometry from three independent experiments showed reduced ubiquitination of K136R. Although several lysine residues are ubiquitinated (Fig. 3*B* and Table 1), these data provide evidence that Lys-136 is a key STAM1 ubiquitination site, which is consistent with the mass spectrometry data. We repeated the ubiquitination reactions in the presence of lysine-less ubiquitin (0K-Ub) which can be attached *via* its C-terminal glycine residue to lysine residues on substrate proteins and is expected to be able to only form polyubiquitin chains *via* its Met-1, since all internal lysine residues are changed to arginine residues. We first compared the conjugation of WT-Ub and 0K-Ub to WT STAM1. Remarkably, 0K-Ub was robustly conjugated to STAM1, although less efficiently than WT-Ub. This is likely due to the restricted number of polyubiquitin chains it can form, since it is only able to form linear polyubiquitinated species once attached to STAM1 (Fig. S1). Importantly, the attachment of 0K-Ub to K136R was reduced when compared with WT STAM1 (Fig. 4*D*). Quantification of either STAM (Fig. 4*E*) or ubiquitin (0K-Ub) (Fig. 4*F*) immunoblots using densitometry from three independent experiments confirmed reduced ubiquitination of K136R, providing further evidence that Lys-136 is a key site for ubiquitin attachment and for linear polyubiquitination. Reactions with WT-Ub (Fig. 4*A*) were incubated for 20 min in order to detect a difference in ubiquitination between WT STAM1 and K136R, in contrast to reactions with 0K-Ub (Fig. 4*E*), which were incubated for 60 min. This difference is likely because of the more robust ubiquitination of STAM1 with WT-Ub than 0K-Ub (Fig. S1) and because attachment of 0K-Ub was partially reduced (Fig. 4*F*), this suggests there are other sites on STAM1 that are modified by linear polyubiquitin chains. Previously, STAM1 has been shown to be modified at its Met-1 (28), thus it is possible this residue and/or other lysine residues are modified with 0K-Ub and linear polyubiquitin chains in our system. Importantly, binding of the K136R variant to βarr1 was similar to WT STAM1 (Fig. 4, G/*H*), indicating that the ubiquitination reaction is not impacted by binding affinity. We did not examine the ability of the STAM1 K136R variant to bind AIP4; however, since the AIP4 binding site does not overlap with Lys-136 (18), it is likely that this binding is not impacted. These data are consistent with the idea that AIP4 attaches an anchor ubiquitin at Lys-136, which is then modified by linear ubiquitin polyubiquitin chains.

**Figure 5:**
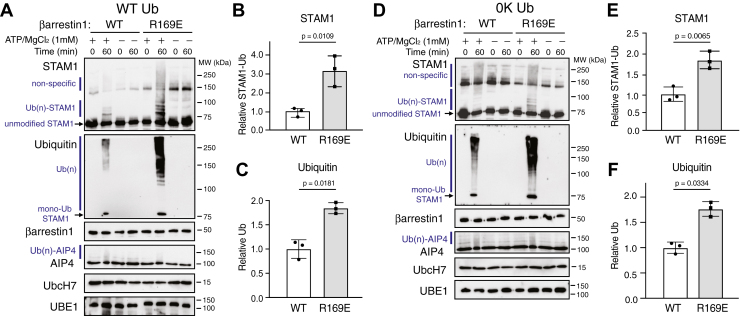
**Analysis of STAM1 ubiquitination by preactivated β-arrestin1.** Ubiquitination reactions as described in Experimental procedures were incubated with 40 nM WT βarr1 (WT) or preactivated variant of βarr1 (R169E) in the presence or absence of 1 mM ATP with WT ubiquitin (WT Ub) (*A*) or lysine-less ubiquitin (0K Ub) (*D*). Reactions were terminated in 2× sample buffer immediately (0 min) or after incubation at 37 °C for 60 min. Reactions were analyzed by 7% SDS-PAGE and immunoblotting. Polyubiquitinated [Ub(n)], unmodified and monoubiquitinated (mono-Ub) STAM1 are indicated. Nonspecific bands are indicated. Data are representative of three independent experiments. STAM1 ubiquitination was quantified from the STAM (*B* and *E*) and ubiquitin (*C* and *F*) immunoblots using densitometry and shown as *bar graphs*. Data were expressed relative to the signal with WT βarr1 and represent the mean ± S.D. from three independent experiments. Data were analyzed by an unpaired *t* test using GraphPad Prism. Adjusted *p* values are indicated. STAM, signal-transducing adaptor molecule.

We previously showed that STAM1 ubiquitination in cells is mediated by GPCR activation (19), suggesting that βarr1 must be activated to mediate STAM1 ubiquitination. To bypass the receptor in our *in vitro* system, we used a variant of βarr1 that is preactivated (R169E) (29). This variant is believed to mimic activated arrestin or arrestin when it is bound to a ligand-activated GPCR (29). When used in the ubiquitination reactions with WT ubiquitin, R169E robustly enhanced the ubiquitination of STAM1 relative to WT βarr1 (Fig. 5*A*). Quantification of either STAM (Fig. 5*B*) or ubiquitin (Fig. 5*C*) immunoblots using densitometry from three independent experiments confirmed enhanced ubiquitination of STAM1. Similarly, when the ubiquitination reactions were performed with 0K-Ub, R169E robustly enhanced linear ubiquitination of STAM1 relative to WT βarr1 (Fig. 5, *D*–*F*). This βarr1 variant did not promote an increase in AIP4 autoubiquitination relative to WT βarr1 (Figs. 5*A* and 7*A*). These data provide further evidence that βarr1 supports linear ubiquitination of STAM1.

**Figure 6:**
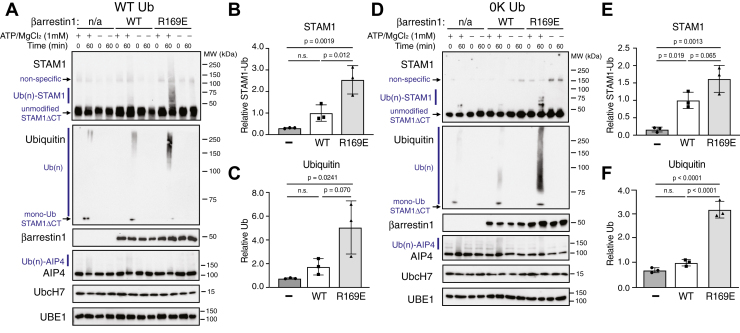
**Evaluation of deleting theC-terminuson STAM1 ubiquitination.** Ubiquitination reactions as described in Experimental procedures were performed with a truncated variant of STAM1 (ΔC-tail) that is still able to bind to β-arr1. Reactions were incubated with 40 nM WT β-arr1 (WT) or preactivated variant of β-arr1 (R169E) or vehicle (n/a) in the presence or absence of 1 mM ATP with WT ubiquitin (WT Ub) (*A*) or lysine-less ubiquitin (0K Ub) (*D*). Reactions were terminated in 2× sample buffer immediately (0 min) or after incubation at 37 °C for 60 min. Reactions were analyzed by 7% SDS-PAGE and immunoblotting. Polyubiquitinated [Ub(n)], unmodified, monoubiquitinated (mono-Ub) STAM1 and nonspecific bands are indicated. Representative immunoblots are shown. STAM1 ubiquitination was quantified from the STAM (*B* and *E*) and ubiquitin (*C* and *F*) immunoblots using densitometry and shown as *bar graphs*. Data were expressed relative to the signal without βarr1 (“−“, minus symbol) and represent the mean ± S.D. from three independent experiments. Data were analyzed by one-way ANOVA GraphPad Prism. Adjusted *p* values are indicated. STAM, signal-transducing adaptor molecule.

To provide additional evidence that STAM1 is modified by linear ubiquitin, we repeated these experiments with a truncated version of STAM1 (1–390; ΔCT) missing the C terminus that we have previously shown is still able to bind βarr1 (30). Similar to full-length STAM1, the βarr1 R169E variant robustly enhanced ubiquitination of ΔCT with WT-Ub relative to reactions that did not have βarr1 or had WT βarr1 (Fig. 6*A*). Quantification of either ΔCT (Fig. 6*B*) or ubiquitin (Fig. 6*C*) immunoblots using densitometry from three independent experiments confirmed enhanced ubiquitination of ΔCT by WT βarr1 and even further by the R169E variant. Similarly, in reactions containing 0K-Ub, ubiquitination of ΔCT was enhanced by the R169E variant relative to WT β-arr1 or reactions without βarr1 (Fig. 6, *D*–*F*), consistent with linear ubiquitin chain modification. Notably, because 0K-Ub is expected to only form linear chains or multi monoubiquitin moieties, the presence of shorter polyubiquitin chains are more obvious relative to reactions with WT-Ub when immunoblotting for ubiquitin from reactions with ΔCT (Fig. 6, *A*/*D*) or WT STAM1 (Fig. 5, *A*/*D*).

**Figure 7:**
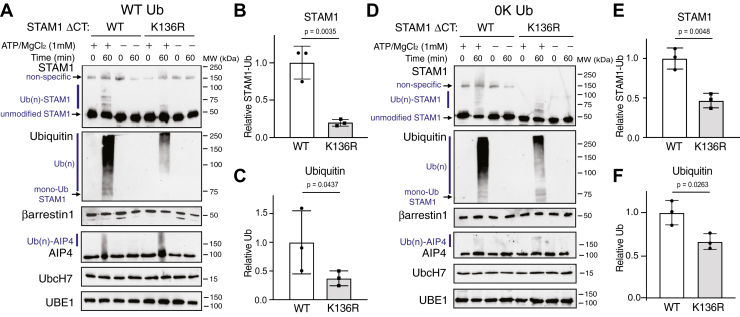
**Evaluation of lysine 136 on STAM1-ΔCT ubiquitination with WT or lysine-less ubiquitin.** Ubiquitination reactions were performed as described in Experimental procedures with STAM1 variant ΔC-tail in which lysine 136 has been substituted with an arginine (K136R). Reactions were performed in the presence of 40 nM preactivated β-arrestin1 and with WT ubiquitin (WT Ub) (*A*) or lysine-less ubiquitin (0K Ub) (*D*). Reactions were performed with or without 1 mM ATP and terminated immediately (0 min) or after incubation at 37 °C for 60 min in 2× sample buffer. Reactions were analyzed by 7% SDS-PAGE and immunoblotting with the indicated antibodies. Polyubiquitinated [Ub(n)], unmodified and monoubiquitinated (mono-Ub) STAM1 are indicated. Representative immunoblots are shown. STAM1 ubiquitination was quantified from the STAM (*B* and *E*) and ubiquitin (*C* and *F*) immunoblots using densitometry and shown as *bar graphs*. Data were expressed relative to the signal from WT ΔCT and represent the mean ± S.D. from three independent experiments. Data were analyzed by ene-way ANOVA GraphPad Prism. Adjusted *p* values are indicated. STAM, signal-transducing adaptor molecule.

To explore this further, we introduced the Lys-136 into the ΔCT background and examined its ubiquitination in the presence of WT-Ub or 0K-Ub. In the presence of WT-Ub, ubiquitination of K136R relative to WT ΔCT was reduced (Fig. 7*A*). Quantification of either ΔCT (Fig. 7*B*) or ubiquitin (Fig. 7*C*) immunoblots using densitometry from three independent experiments confirmed reduced ubiquitination of K136R ΔCT relative to WT. Similarly, in the presence of 0K-Ub, ubiquitination of K136R relative to WT ΔCT was reduced (Fig. 7*D*), which was confirmed by quantification from three independent experiments (Fig. 7, *E*/*F*). Importantly, the degree of reduced ubiquitination observed with ΔCT-K136R (Fig. 7) was greater than that observed with WT STAM1 (Fig. 5), suggesting that the C-tail of STAM1 may play a regulatory role in STAM1 ubiquitination. These data provide additional evidence that βarr1 supports linear ubiquitination of STAM1 at Lys-136.

**Figure 8:**
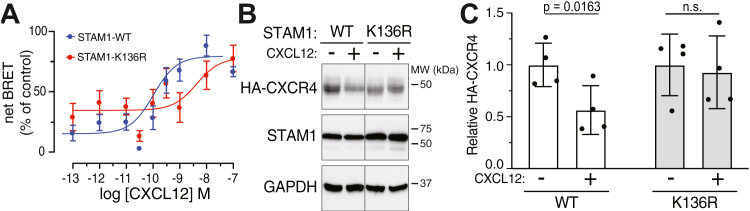
**BRET analysis between STAM K136R and β-arrestin1, degradation assays.***A*, HEK293 cells cotransfected with donor plasmid (Rluc8-WT STAM1 or Rluc8-STAM1-K136R) and acceptor plasmid (β-arr1-WT-GFP^10^) were stimulated with increasing concentrations of CXCL12 for 2 min before DeepBlueC was added. BRET measurements were taken 30 min after the addition of the luciferase substrate. Curves were fitted by nonlinear regression, assuming a single binding site (GraphPad Prism). Data represent the mean ± S.E.M. net BRET from three independent experiments. *B*, HEK293 cells transiently expressing HA-CXCR4 and either WT-STAM or K136R-HIS-tagged STAM treated with cycloheximide were stimulated with either vehicle (“−“, minus symbol) or 10 nM CXCL12 for 3 h. Equal amounts of lysates were analyzed by 10% SDS-PAGE and immunoblotting. Representative immunoblots are shown. *Vertical line* indicates that intervening lanes were removed from representative immunoblots from the same exposure. *C*, CXCR4 degradation was quantified using densitometry and shown as *bar graphs*. After normalizing to the signal from loading control GADP, data were expressed relative to the signal from vehicle (“−“, minus symbol) and represent mean ± S.D. from four independent experiments. Data were analyzed by one-way ANOVA, followed by Tukey’s post hoc test. Adjusted *p* values are indicated. BRET, bioluminescence resonance energy transfer; Rluc, Renilla luciferase; STAM, signal-transducing adaptor molecule.

Previously, we showed that βarr1 and STAM1 form a complex in cells (19, 22). To explore the role of Lys-136 in cells, we examined whether the STAM1 K136R variant is able to bind to βarr1 in cells following stimulation of CXCR4 with its cognate ligand, CXCL12. For this, we performed bioluminescence resonance energy transfer (BRET) experiments with FLAG-tagged CXCR4 transiently coexpressed in HEK293 cells with Renilla luciferase (Rluc8)-STAM1 (WT or K136R variant) and βarr1-GFP, as we have previously described (17). As expected, CXCL12 stimulation promoted a dose-dependent increase in the BRET response between βarr1-GFP and WT Rluc8-STAM1 (Fig. 8*A*), consistent with STAM1 recruitment to βarr1. In contrast, the BRET response between βarr1-GFP and variant Rluc8-STAM1-K136R was shifted to the right, suggesting reduced binding between STAM1-K136R and βarr1. Because Lys-136 is modified by linear ubiquitination, these data suggest that linear polyubiquitin at Lys-136 impacts the stability of the STAM1:βarr1 complex in cells.

Previously, we have shown that the STAM1:βarr1 complex regulates lysosomal trafficking of the chemokine receptor CXCR4 (19). We examined the role of Lys-136 on agonist-stimulated degradation of CXCR4, an indication of receptor trafficking to a terminal lysosomal degradative compartment, as we have previously shown (19). In cells expressing WT STAM1, HA-tagged CXCR4 was robustly degraded following agonist stimulation (Fig. 8, *B*/*C*). In contrast, in cells expressing K136R-STAM, agonist-stimulated HA-CXCR4 degradation was essentially blocked (Fig. 8, *B*/*C*), suggesting this variant acts as a dominant-negative in cells and blocks lysosomal trafficking of CXCR4. These data suggest that linear ubiquitination at Lys-136 on STAM1 is required for proper lysosomal trafficking of CXCR4.

## Discussion

In this study, we reconstituted STAM1 ubiquitination *in vitro* and provide evidence that βarr1 is an adaptor for STAM1 ubiquitination by AIP4. Mass spectroscopy analysis indicates that STAM1 is modified at several internal lysine residues and with several types of polyubiquitin chains. Specifically, we provide evidence that Lys-136 on STAM1 represents a key AIP4 ubiquitination site and serves as an anchor for conjugation by linear ubiquitin chains, which we provide evidence is directed by βarr1. To our knowledge, this is the first time linear ubiquitin linkage has been associated with an E3 ligase other than LUBAC (23). Our data suggest that βarr1 specifies linear polyubiquitin chains, although it may not necessarily be responsible for lysine selection or attachment of the anchor ubiquitin on STAM1. We also provide evidence suggesting this modification is required for GPCR lysosomal trafficking. Our study furthers our understanding of βarrestins as E3 ligase adaptors and expands the roles of linear polyubiquitin in cell biology.

A major finding of our study is that we provide evidence that STAM1 is modified by linear polyubiquitin chains by the E3 ligase AIP4. To date, LUBAC is the only E3 ligase complex known to conjugate linear polyubiquitin chains (31). Linear or M1-linked polyubiquitin chains are formed when the C-terminal Gly-76 of one ubiquitin is conjugated to the amino group of the Met-1 residue in the preceding ubiquitin. LUBAC attaches linear chains to an anchor ubiquitin that is provided or primed by another E3 (32). In our system, the anchor ubiquitin on STAM1 is provided by AIP4, which our data indicate is placed on Lys-136 and likely other residues (Figs. 4 and 7). βarr1 may not be required for attachment of the anchor ubiquitin at Lys-136 because we detected modification of this site in the absence of βarr1 by mass spectrometry (Fig. 3). Therefore, it is possible that βarr1 specifies the attachment of linear polyubiquitin chains beginning at the anchor ubiquitin. The location of Lys-136 on the Vps27, HRS, and STAM domain of STAM1 and βarr1 binding mainly to the helical region of STAM1 could facilitate the positioning of AIP4 in a way that favors ubiquitination of this lysine residue by Met-1 ubiquitin linkages. Because STAM1 is ubiquitinated at its own Met-1 (28), it is possible that structural restrictions prevent βarr1 from facilitating AIP4-mediated modifications at this site. The side chain of solvent-exposed Lys-136 is located on the opposite end of the Vps27, HRS, and STAM domain and is oriented directly away from Met-1 (33). The remaining polyubiquitinated species observed with K136R-STAM1 (Fig. 4) or K136R-ΔCT (Fig. 7) in the presence of 0K-Ub is likely due to polyubiquitin chains formed at Met-1 of STAM1 or other internal lysine residues, potentially independent of βarr1. Our data suggest that βarr1 is an adaptor that directs linear polyubiquitin ubiquitin chains at Lys-136.

In addition to linear ubiquitin, our data reveal that STAM1 is also modified by K27, K29, K33, K48, and K63-linked polyubiquitin chains, which are chain linkages previously associated with AIP4 (21). In addition, we also observed K-11 chains in the ubiquitination reactions (Table 1). These linkages are likely from conjugation to STAM1 at one of the seven internal lysine residues we identified to be modified by ubiquitin (Table 1). The lysine residues we identified overlap with previously identified ubiquitinated sites on STAM1 from proteomic screens for ubiquitinated proteins, except for Lys-346 (28, 34). We did not observe ubiquitination of βarr1 by mass spectrometry or by Western blotting, suggesting that although it is recognized by AIP4, βarr1 is not a substrate. In addition, AIP4 autoubiquitination was detected in our *in vitro* system (Figs. 4 and 5), although we did not detect which lysine residues are modified by mass spectrometry. Overall, our mass spectrometry analysis reveals a previously unknown linkage (K11) by AIP4 and a novel ubiquitination site on STAM1 (Lys-346), in addition to linear polyubiquitin chains. It remains unclear if modifications at these sites are dependent upon βarr1 or GPCR activation. These data highlight the diversity and complexity of the ubiquitin code.

We provide evidence that βarr1 facilitates STAM1 ubiquitination by AIP4 by acting as an adaptor between STAM1 and AIP4. STAM1 binds to the finger-loop region of βarr1 (14), and we show that a STAM1-deficient binding variant of βarr1 does not support STAM1 ubiquitination (Fig. 2*A*). AIP4 binds to a different surface on βarr1 (16), and its binding to this variant remains intact (Fig. 2, *D*/*E*). We also show that STAM1 ubiquitination is enhanced in the presence of preactivated βarr1 (Figs. 5 and 6) consistent with our previous cell-based experiments showing STAM1 ubiquitination is enhanced by GPCR stimulation (19). This also correlates with enhanced binding of STAM1 and AIP4 to βarr1 upon GPCR stimulation (16). AIP4 typically exists in an autoinhibitory conformation and is activated for substrate ubiquitination by phosphorylation (35) or by binding to adaptor proteins (21). AIP4 autoubiquitination occurs in both the presence of WT and preactivated βarr1 (Figs. 4 and 5), suggesting that βarr1 activates AIP4. However, this activation was modest in our system, suggesting that reaction conditions are optimized for STAM1 ubiquitination rather than AIP4 autoubiquitination. Thus, in addition to an adaptor role, βarr1 is also likely activating AIP4 to complete STAM1 ubiquitination. Overall, our *in vitro* reconstituted ubiquitination system mirrors STAM1 ubiquitination observed in cells.

Previously, we linked βarr1 binding to STAM1 to CXCR4 lysosomal trafficking (19). Our data here reveal that while binding of K136R-STAM1 to βarr1 remains intact *in vitro* (Fig. 4*C*), in cells this variant shows reduced binding to βarr1 following CXCR4 activation by its cognate agonist (Fig. 8*A*). These data suggest that linear polyubiquitination at Lys-136 of STAM1 is required to stabilize the STAM1:β-arr1 complex (Fig. 8*A*). When expressed in cells, K136R-STAM1 acts as a dominant negative, and attenuates agonist-stimulated CXCR4 degradation (Fig. 8, *B*/*C*), suggesting that linear ubiquitination at Lys-136 is required for proper lysosomal trafficking of this GPCR. Previously, we showed that expression of minigenes that disrupt the βarr1:STAM1 complex accelerates CXCR4 degradation (19). However, the minigenes affect the ubiquitination status of other endosomal sorting complexes required for transport proteins as well and might have a broader impact on the endosomal sorting complexes required for transport pathway in a manner that favors GPCR lysosomal trafficking (19). The K136R variant more directly addresses the role of STAM1 and by extension linear ubiquitination on CXCR4 trafficking. How linear ubiquitination might impact the stability of the βarr1:STAM1 complex and precisely impact GPCR trafficking remains unclear, although it is interesting that linear polyubiquitin chains have previously been shown to bind to ubiquitin binding domains of STAM1 with relatively high-affinity (33). Whether this feature is linked to the stability of the βarr1:STAM1 complex remains to be determined. Attempts to detect linear-polyubiquitin chains at Lys-136 in cells using several commonly applied biochemical approaches (27, 36) have so far been inconclusive and will require experimental optimization and tool development. In addition, we have previously shown that the βarr1:STAM1 complex mediates focal adhesion kinase signaling and chemotaxis by CXCR4 (22), but whether linear ubiquitination of STAM1 plays a role in CXCR4 signaling remains to be determined. Nevertheless, our data suggest that linear polyubiquitination is linked to the stability of the β-arr1:STAM1 complex and to GPCR trafficking.

In summary, we show that βarr1 serves as a linear polyubiquitination adaptor for the effector molecule STAM1 by the E3 ligase AIP4. LUBAC is the only E3 ligase known to conjugate linear polyubiquitin chains (31), and many previously described physiological roles of linear polyubiquitin have been attributed to LUBAC in the context of proinflammatory signaling (29). We provide evidence that linear ubiquitin may have additional roles beyond those previously described, including within the context of GPCR trafficking.

## Experimental procedures

### Chemicals, reagents, and antibodies

The rabbit monoclonal antibodies against STAM1 (cat. #12434-1-AP) and Ube1 (cat # 15912-1-AP) and mouse monoclonal antibody against GAPDH (cat. #60004–1) were from Proteintech. The rabbit monoclonal antibodies against UbcH7 (cat. #8721), ubiquitin (cat. #43124), STAM1 (cat #13053), and βarrestin1 (cat. #12697) were from Cell Signaling Technology. The rabbit monoclonal antibody against AIP4 (cat. #ab108515) was from Abcam. The mouse monoclonal antibody against the His epitope (cat. #A00186) was from GenScript. The mouse monoclonal antibody against the hemagglutinin epitope was from BioLegend (cat. # MMS-101R). ATP (cat #A2383-5G) was from MilliporeSigma. UbcH7 (E2; cat# E2-640–100), lysine-less ubiquitin (0K-Ub; cat# UM-NOK) and lysine-less biotinylated ubiquitin (0K-Ub biotin; cat# UB-560) were from R&D Systems. ATP (cat# 34369–07–8) and MgCl_2_ (cat# M8266) were from Sigma-Aldrich. Protease inhibitors aprotinin (cat# 97062–754), leupeptin (cat # 97063–924), pepstatin (cat # 97064–248), and pH test strips (cat# BDH35309.606) were from VWR.

### DNA plasmids

Bacterial expression plasmids for βarr1 (37), βarr1-4A (17), and GST-AIP4 (20) were previously described. Dr Francis Peterson (Medical College of Wisconsin) generously provided pET15-His-SUMO plasmid. New plasmids were generated using standard PCR conditions for quick change site-directed mutagenesis and DNA assembly using NEBuilder HiFi DNA Assembly Kit (New England Biolabs, cat #E5520S). The cloning information is provided in Table S1. All plasmids were transformed into NEB 5-alpha competent cells (cat # C2987H) and verified by dideoxy sequencing.

### Protein expression and purification

Purified, untagged ubiquitin used for mass spectrometry experiments was purified as previously described (38) or was a kind gift from Samuel DeCero and Dara Frank (Medical College of Wisconsin), which was purified as previously described (39). Untagged βarrestin1, βarrestin1-4A, and βarrestin1-R169E were purified as previously described (17). GST-AIP4 was purified as previously described (20), and the GST tag was removed *via* incubation with HRV3C protease in 20 mM Tris 150 mM NaCl pH 7.5 (TBS) at 4 °C.

pET28a-His-UBE1 plasmid was a kind gift from Matthew Scaglione (Duke University). The plasmid was expressed in BL21 DE3 cells, induced with 1 mM IPTG overnight at 16 °C, and cell pellets were lysed in Tris-buffer 1 (50 mM Tris pH 8.0, 150 mM NaCl, 1 mM EDTA-NaOH pH 8.0, 0.1% v/v Triton-X100 plus 1 mM aprotinin, 1 mM leupeptin, and 1 mM pepstatin), sonicated and clarified by centrifugation. The supernatant was incubated with nickel resin (Thermo Fisher Scientific; cat# 88221) for 1 h at 4 °C while rocking. The resin was washed twice with wash buffer 1 (50 mM sodium phosphate buffer pH 8.0, 300 mM NaCl, then washed once with wash buffer 2 (50 mM sodium phosphate buffer pH 8.0, 150 mM NaCl. His-UBE1 was eluted from the resin with 200 mM imidazole in 50 mM sodium phosphate buffer, concentrated to a volume of 10 ml using an Amicon Ultra 0.5 centrifugal concentrator, followed by chromatography *via* Q-Sepharose (GE Life Science) column. His-UBE1 was eluted from the Q column with 50 mM sodium phosphate and 150 mM NaCl. Purity was verified by SDS-PAGE, and the concentration was determined *via* bicinchoninic acid (BCA) assay with bovine serum albumin (BSA) standards (Thermo Fisher Scientific; cat #23208) and Pierce 660 nM protein assay reagent (Thermo Fisher Scientific; cat #22660).

pQE30-STAM-His plasmids coding for STAM1 with modified N-terminal amino acid sequence (Met-Arg-Gly-Ser preceding Pro-2, UniProt Q92783) were expressed in BL21 DE3 cells, induced with 1 mM IPTG for 18 h at 18 °C and cell pellets were lysed in Tris-buffer 2 (20 mM Tris–HCl pH 7.5, 300 mM NaCl, 0.1% v/v Triton X100, 10 mM imidazole, 1 mg/ml lysozyme, 1 mM aprotinin, 1 mM leupeptin, and 1 mM pepstatin), sonicated, and clarified by centrifugation. The supernatant was incubated with TALON cobalt resin (Takara; cat# 635502) for 3 h at 4 °C while rocking. The resin was washed twice with Tris-buffer 2 without Triton-X100, followed by elution of STAM-His with 200 mM imidazole in Tris-buffer 2 without Triton-X100. Purity was verified by SDS-PAGE, and concentration was determined *via* BCA assay with BSA standards.

pET-SUMO-STAM-His and pET-SUMO-ΔC-tail-STAM-His plasmids were expressed in Rosetta cells and expression was induced with 0.5 mM IPTG for 3 h at 37 °C and cell pellets were lysed in Tris-buffer 3 (20 mM Tris pH 7.5, 150 mM NaCl, 0.1% v/v Triton X-100, 0.1% β-mercaptoethanol, 1 mM PMSF, 1 mM aprotinin, 1 mM pepstatin, and 1 mM leupeptin, and 1 mM benzamidine) sonicated and clarified by centrifugation. The supernatant was incubated with TALON cobalt resin for 2 h at 4 °C while rocking. The resin was washed twice with TBS, followed by elution of SUMO-STAM-His with 200 mM imidazole in TBS. Eluted protein was pooled, treated with ULP1 protease to cleave the SUMO tag and leave the first methione of STAM1 intact, and dialyzed in TBS. The dialyzed and cleaved protein was then incubated with TALON cobalt resin for 1 h at 4 °C, then subjected to a reverse cobalt column to separate STAM-His from all other protein components. Purity was verified by SDS-PAGE, and concentration was determined *via* BCA assay with BSA standards.

### *In vitro* ubiquitination assay

Reactions contained E1 (Ube1; 42 nM), E2 (UbcH7; 350 nM), and E3 (AIP4; 48.5 nM), ubiquitin (WT or lysine-less; 11.6 μM), STAM1 (WT or K136R; 42 nM), βarrestin1 (WT, βarr1-4A, or βarr1-R169E; 40 nM), 1 mM DTT, and 1 mM ATP/MgCl_2_. For all reactions, ATP/MgCl_2_ 1M solution was made fresh at ∼pH 7, determined using pH test strips. Reactions were incubated for 20 or 60 min at 37 °C. For Figure 1, βarrestin1 concentrations were 0 nM, 20 nM, 40 nM, 80 nM, 160 nM, or 320 nM and reactions were incubated at 37 °C for 90 min. Reactions for mass spectrometry were performed with C-terminal HIS-tagged STAM1 containing the following N-terminal amino acid sequence before Pro-2 (UniProt: Q92783): Met-Arg-Gly-Ser. Reactions were quenched with equal volumes of 2 × sample buffer (8% SDS, 1% glycerol, 37.5 mM Tris–HCl, pH 6.5, 0.7 M β-mercaptoethanol, 0.000009% bromophenol blue). Samples were analyzed by 7% or 10% SDS-PAGE, immunoblotting, and imaging with enhanced chemiluminescence horseradish peroxidase SuperSignal west dura extended duration substrate (Thermo Fisher Scientific, cat# 34075). Reactions for Figure 1 used the anti-STAM1 antibody from Cell Signaling, while reactions for all other Figures used the anti-STAM1 antibody from ProteinTech. STAM1 ubiquitination was quantified using densitometry-based quantification (Image J, NIH) of ubiquitinated species from anti-STAM1 and anti-ubiquitin immunoblots and plotted using GraphPad Prism.

### Pull-down assays

Purified βarrestin1 (0.1 μM, 0.14 μg) was incubated with WT or K136R STAM1-His (0.3 μM, 0.8 μg) in 50 μl TBS for 20 min at 37 °C. To isolate complexes, 20 μl of 50/50 slurry of TALON cobalt resin (Takara cat# 635502) equilibrated with TBS binding buffer (20 mM Tris–HCl pH 7.5, 150 mM NaCl) was added to the protein mixture. Samples were gently rocked at 4 °C for 1 h, washed three times with ice cold TBS and proteins were eluted in 50 μl 250 mM imidazole while rocking for 15 min at room temperature. For GST pull-down assays, BL21 cells transformed with GST-AIP4 or GST empty vector (pGEX-4T2) were induced with 0.1 mM IPTG for 4 h at 18 °C, as we have previously described (40). Cell pellets were lysed in binding buffer (20 mM Tris–HCl pH 7.5, 150 mM NaCl, 0.1% v/v Triton X-100, 1 mM DTT, and protease inhibitor cocktail (Sigma-Aldrich, P8340)) and proteins were purified by immobilization on glutathione 4B-Sepharose resin, as we have previously described (18). WT βarr1 or βarr1-4A (100 nM) were incubated with equimolar (600 nM) immobilized GST or GST-AIP4 while rocking at 4 °C for 2 h, washed 3 times with binding buffer, and proteins were eluted in 2 × sample buffer. Eluted and input samples were resolved by 10% SDS-PAGE, analyzed by immunoblotting and imaging with enhanced chemiluminescence horseradish peroxidase SuperSignal West Dura extended duration substrate. βarrestin binding was quantified using densitometry-based quantification (Image J, NIH) and plotted using GraphPad Prism, version 10.0.0 for Macintosh (www.graphpad.com).

### Mass spectrometry analysis of STAM1 ubiquitination

Three separate mass spectrometry experiments were performed. In the first experiment, *in vitro* ubiquitination experiments were performed as described above, except that βarr1 and STAM1 were preincubated for 30 min at 37 °C, before adding a mixture of complex to reactions. Ubiquitination reactions were trypsin digested and analyzed by liquid chromatography tandem mass spectrometry and peptide identification. In the second and third mass spectrometry experiments, 20 μl of cobalt resin was added to samples and incubated for 1 h at 4 °C to precipitate C-terminal His-tagged STAM. Samples were subject to on-bead trypsin digestion and analyzed by liquid chromatography tandem mass spectrometry and peptide identification. A detailed protocol for mass spectrometry is included in Supplemental methods and Tables S2 and S3. Mass spectrometry data are provided in File S1. Mass spectrometry was performed by the Center for Biomedical Mass Spectrometry Research, Medical College of Wisconsin.

### BRET assay

To measure GPCR-stimulated βarr1 binding to WT STAM1 or the K136R variant, HEK293 cells grown on 10-cm dishes were cotransfected with FLAG-tagged CXCR4 (2 μg), Rluc8-STAM1 (200 ng; WT or K136R), and βarr1-GFP^10^ (600 ng) using PEI (Polysciences, Ic, cat# 23966), as we have previously described (17). The next day, cells were seeded into 96-well white microplates with clear bottom in Dulbecco's modified Eagle's medium (DMEM) containing 5% fetal bovine serum at a density of 20,000 cells/well. The following day, cells were washed with Hank’s balanced salt solution containing 20 mM Hepes, pH = 7.4 and then replaced with 80 μl of the same buffer. Cells were stimulated with increasing concentrations of CXCL12 (Protein Foundry) for 15 min at 37 °C, 5% CO_2_. After incubation with ligand, 5 μM luciferase substrate coelenterazine 400A (DeepBlue C) was added. White tape was adhered to the bottom of the plate and BRET measurements were performed with a BMG Labtech LUMIstar OMEGA microplate reader. Rluc donor emission was detected at 440 ± 40 nm and acceptor GFP emission was detected at 515 nm ± 15 nm bandwidth. For each BRET experiment, cells transfected with only the Rluc plasmid were included as an internal background control. BRET ratios were calculated by dividing the acceptor emission light intensity by the donor emission light intensity, as we previously described (17). BRET ratios from the first three consecutive reads were averaged. BRET was determined by subtracting BRET from cells expressing only Rluc plasmid from BRET for each ligand dose to generate net BRET.

### Degradation assay

HEK293 cells grown in 10 cm dishes were transfected with HA-CXCR4 (0.5 μg) and either WT His-tagged STAM (9 μg) or K136R (9 μg) using PEI. The next day, cells were passaged onto 6-well plates. Twenty-four hours later, cells were washed once with DMEM and then incubated with DMEM containing 10% fetal bovine serum and 50 μg/ml cyclohexamide for 15 min at 37 °C, followed by stimulation with vehicle (sterile water) or 10 nM CXCL12 for 3 h. Cells were washed once with 500 μl PBS and collected in 300 μl of 2× sample buffer. Samples were analyzed by 10% SDS-PAGE and immunoblotting with antibodies against the HA-tag for HA-CXCR4, GAPDH, and STAM1. Receptor degradation was determined by densitometric analysis of immunoblots from multiple experiments. Degradation was calculated as the percentage receptor degraded in CXCL12-treated cells compared with vehicle.

### Statistical analysis

Data comparing only two means were analyzed by unpaired *t* test. Data comparing more than two means were analyzed by one-way analysis of variance (ANOVA) with Tukey’s post hoc test using Prism 10 for Macintosh (GraphPad).

## Data availability

All data associated with this study are contained within the article.

## Supporting information

This article contains supporting information.

## Conflict of interest

The authors declare that they have no conflicts of interests with the contents of this article.

## Supporting information

plasmid table McElrath et al 2023
