# Supplementary material for: β-arrestin1 is an E3 ubiquitin ligase adaptor for substrate linear polyubiquitination
Source: J Biol Chem. 2023 Nov 21;299(12):105474. doi: 10.1016/j.jbc.2023.105474 (PMC10755771; doi:10.1016/j.jbc.2023.105474)
Supplement: Supplemental Methods [file mmc3.docx]

**Sample Digestion**

After adding 100µl of ammonium bicarbonate, the beads were transferred to a 1.5ml tube and centrifuged 5 minutes at 14000×g. The supernatant was removed, and the beads were washed again with 100µl and centrifuged again 5 min at 14,000×g. The beads were resuspended in 100 µl of 100mM ammonium bicarbonate and 40% (2x) Invitrosol. Cysteines were reduced in 5mM TCEP for 30 min at 37°C and alkylated with 10 mM iodoacetamide for 30 min at 37°C. The proteins were digested overnight with 5 µg of trypsin at 37$^{\circ}$C. After cleanup by SP2, the Pierce Quantitative Fluorescent Peptide Assay (Thermo Scientific) was used to determine peptide concentrations. All samples were then diluted to 20 ng/µl.

**Mass Spectrometry Analysis**

Each sample was analyzed on a Thermo Scientific Orbitrap Fusion Lumos MS via 2 technical replicate injections using a data-dependent acquisition (DDA) HCD MS2 instrument method outlined in Table S2.

MS data were analyzed using Proteome Discoverer 2.3 (Thermo) platform as outlined in the Table S3. Protein identifications were filtered to include only those proteins identified by two or more unique peptides identified and ranked as high confidence.

| **Table S2. Chromatography and MS instrument acquisition settings** | | | |
| --- | --- | --- | --- |
| ***Sample Volume*** | 10 µL | ***Isolation Window*** | 1.6 m/z |
| ***Stationary Phase*** | Thermo Acclaim PepMap C_18_  75µm × 50cm | ***MS^2^ AGC Target*** | 5e4 |
| ***LC Solvent A*** | 100% H_2_O,  0.1% formic acid | ***MS^2^ Maximum IT*** | 54 ms |
| ***LC Solvent B*** | 80% acetonitrile,  0.1% formic acid | ***Normalized Collision Energy*** | 30 |
| ***Gradient Ramp and***  ***Duration***  ***Flow Rate*** | 2.5-5% B in 1 minute  5-7% B in 4 minutes  7-28% B in 72 minutes  28-60%B in 10 minutes  60-99%B in 2 minutes  300 nL/min | ***Minimum Intensity Req.*** | 5e5 |
| ***Mass Spectrometer*** | Thermo Orbitrap Fusion Lumos | ***Dynamic Exclusion*** | 60.0 s |
| ***Spray Voltage*** | 2.1 kV | ***MS^2^ acquisition*** | Data dependent, 3 s cycle time, Centroid |
| ***In-Source CID*** | 0.0 eV | ***MS^2^ Fragmentation*** | HCD |
| ***MS^1^ scan range*** | 375-1500 m/z | ***MS^2^ Detection*** | Orbitrap |
| ***MS^1^ resolution*** | 120,000 @ 200 *m/z* | ***MS^2^ fixed first mass*** | 110 *m/z* |
| ***MS^1^ AGC Target*** | 4e5 | ***MS^2^ resolution*** | 30,000 @ 200 *m/z* |
| ***MS^1^ Maximum IT*** | 50 ms | ***Advanced Precursor Determination*** | on |

| **Table S3. Mass spectrometry data processing parameters** | | | |
| --- | --- | --- | --- |
| ***Platform*** | ProteomeDiscoverer 2.3 | ***Precursor mass tolerance*** | 10 ppm |
| ***Search Algorithms*** | SequestHT | ***Fragment mass tolerance*** | 0.02 Da |
| ***Validation*** | Percolator  Peptide Validator  Protein FDR Validator | ***Target FDR (Strict) for PSMs:*** | 0.01 |
| ***Database*** | Swissprot Human; created 05/01/2019 | ***Target FDR (Relaxed) for PSMs:*** | 0.05 |
| ***Digest*** | Trypsin (semi)  3 Missed Cleavages Allowed | ***Target FDR (Strict) for Peptides:*** | 0.01 |
| ***Static Modifications*** | Carbamidomethyl (C) | ***Target FDR (Relaxed) for Peptides:*** | 0.05 |
| ***Dynamic Modifications*** | Gly-Gly (K),  Oxidation (M), Acetylation (protein N-terminus) |  |  |
