# Supplementary material for: β-arrestin1 is an E3 ubiquitin ligase adaptor for substrate linear polyubiquitination
Source: J Biol Chem. 2023 Nov 21;299(12):105474. doi: 10.1016/j.jbc.2023.105474 (PMC10755771; doi:10.1016/j.jbc.2023.105474)
Supplement: Figure S1 [file mmc4.pdf]

Supplemental Figure S1. McElrath et. al.

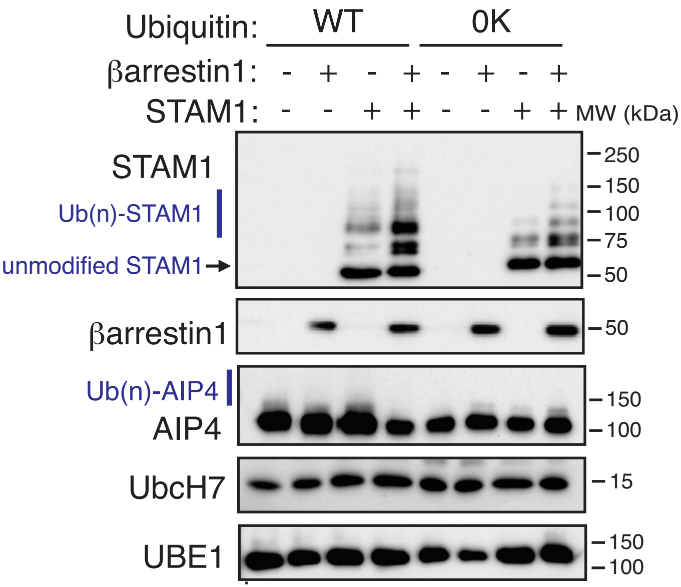

Figure S1. STAM1 ubiquitination with wild-type (WT) or lysine-less ubiquitin (0K). STAM1 ubiquitination by AIP4 was reconstituted *in vitro* and performed in the presence of either WT or 0K ubiquitin and in the presence or absence of βarrestin1. Ubiquitination reactions contained E1(Ube1, 42 nM), E2 (UbcH7, 350 nM), E3 (AIP4, 97 nM), βarrestin1 (150 nM), ubiquitin (11.6 μM), ATP/MgCl2 (10 μM) plus STAM1 (42 nM) in 40 μL. STAM1 and βarrestin1 were incubated together at 37˚C for 30 minutes prior to the start of the reaction. After the addition of ATP/MgCl2, reactions were incubated for 90 minutes at 37˚C and terminated with 40 μL 2x sample buffer. Samples were boiled at 100˚C for 5 minutes and equal volumes were analyzed by 7% SDS-PAGE and immunoblotting with the indicated antibodies. Poly-ubiquitinated [Ub(n)] and unmodified STAM1 are indicated. Immunoblots are from one representative experiment performed independently four times.
